# Use of photostable green fluorescent protein mStayGold for live-cell imaging in *Dictyostelium discoideum*

**DOI:** 10.17912/micropub.biology.002300

**Published:** 2026-10-13

**Authors:** Hidenori Hashimura, Shoko Fujishiro, Toyoko Sugita, Hibiki Nakagawa, Satoshi Sawai

**Affiliations:** 1 Graduate School of Arts and Sciences, The University of Tokyo, Meguro-ku, Tokyo, Japan; 2 Research Center for Complex Systems Biology, Universal Biology Institute, The University of Tokyo, Meguro-ku, Tokyo, Japan

## Abstract

*Dictyostelium discoideum* is a key Amoebozoa model organism in cell biology, offering unique insights into chemotaxis, cell polarity and the transition from unicellular to multicellular life. Here, we evaluated the highly photostable fluorescent protein mStayGold for live-cell imaging in *D. discoideum* . mStayGold showed localization patterns comparable to Achilles when fused to Lifeact or the PKBR1(N150) membrane marker. Compared with Achilles, mStayGold exhibited greater resistance to photobleaching under continuous illumination and lower cell-to-cell variability in fluorescence intensity. These properties make mStayGold a promising probe for demanding imaging applications, including super-resolution microscopy, high-speed imaging, long-term developmental imaging and single-molecule tracking.

**Figure 1. Expression of mStayGold in D. discoideum:** (A) Cells expressing mStayGold under the *act15* promoter. Vegetative cells (upper panel) and slug-stage cells (lower panel). Scale bar, 10 µm. Brightfield (left panels) and fluorescence images (right panels). (B–D) Snapshots of vegetative (upper panel) and slug-stage (lower panel) cells expressing *act15p* :mStayGold-Lifeact, *coaAp* :PKBR1(N150)-mStayGold and *coaAp* :PH _Akt_ -mStayGold (left, brightfield channel; right, fluorescence channel). Scale bar, 10 µm. The anterior-posterior axis of the slug is from left to right (A–D). (E) Vegetative cells expressing PKBR1(N150)-Achilles (top), PKBR1(N150)-mNeonGreen (middle) and PKBR1(N150)-mStayGold (bottom) under the *coaA* promoter (left, brightfield channel; right, fluorescence channel). Scale bar, 100 µm. (F) Violin plot of the single-cell mean fluorescence intensity distribution of PKBR1(N150)-Achilles, PKBR1(N150)-mNeonGreen and PKBR1(N150)-mStayGold-expressing vegetative cells (PKBR1(N150)-Achilles, n = 189 cells. PKBR1(N150)-mNeonGreen, n = 196 cells. PKBR1(N150)-mStayGold, n = 258 cells). The black line indicates the median. **: *P* < 10 ^−4^ . *: *P* < 0.05. NS: not significant ( *P* > 0.05). (G) Slugs expressing PKBR1(N150)-Achilles (top), PKBR1(N150)-mNeonGreen (middle) and PKBR1(N150)-mStayGold (bottom) under the *coaA* promoter (left, brightfield channel; right, fluorescence channel). The anterior-posterior axis of the slug is from left to right. Scale bar, 100 µm. (H) A violin plot of the mean fluorescence intensities in the anterior and posterior regions of the slugs (PKBR1(N150)-Achilles, n = 10 slugs. PKBR1(N150)-mNeonGreen, n = 10 slugs. PKBR1(N150)-mStayGold, n = 10 slugs). The black line indicates the median value. Statistical comparisons were performed between the three fluorescent proteins within the anterior and posterior regions separately. *: *P* < 10 ^−3^ . NS: not significant ( *P* > 0.05). (I) Fluorescence images of slug-stage cells expressing mStayGold-Lifeact (upper panel) and Achilles-Lifeact (lower panel). The anterior-posterior axis of the slug is from left to right. Scale bar, 10 µm. (J) Time-course plot of the normalized fluorescence intensity of Achilles-Lifeact and mStayGold-Lifeact in slugs (mStayGold-Lifeact, n = 3 slugs. Achilles-Lifeact, n = 3 slugs).

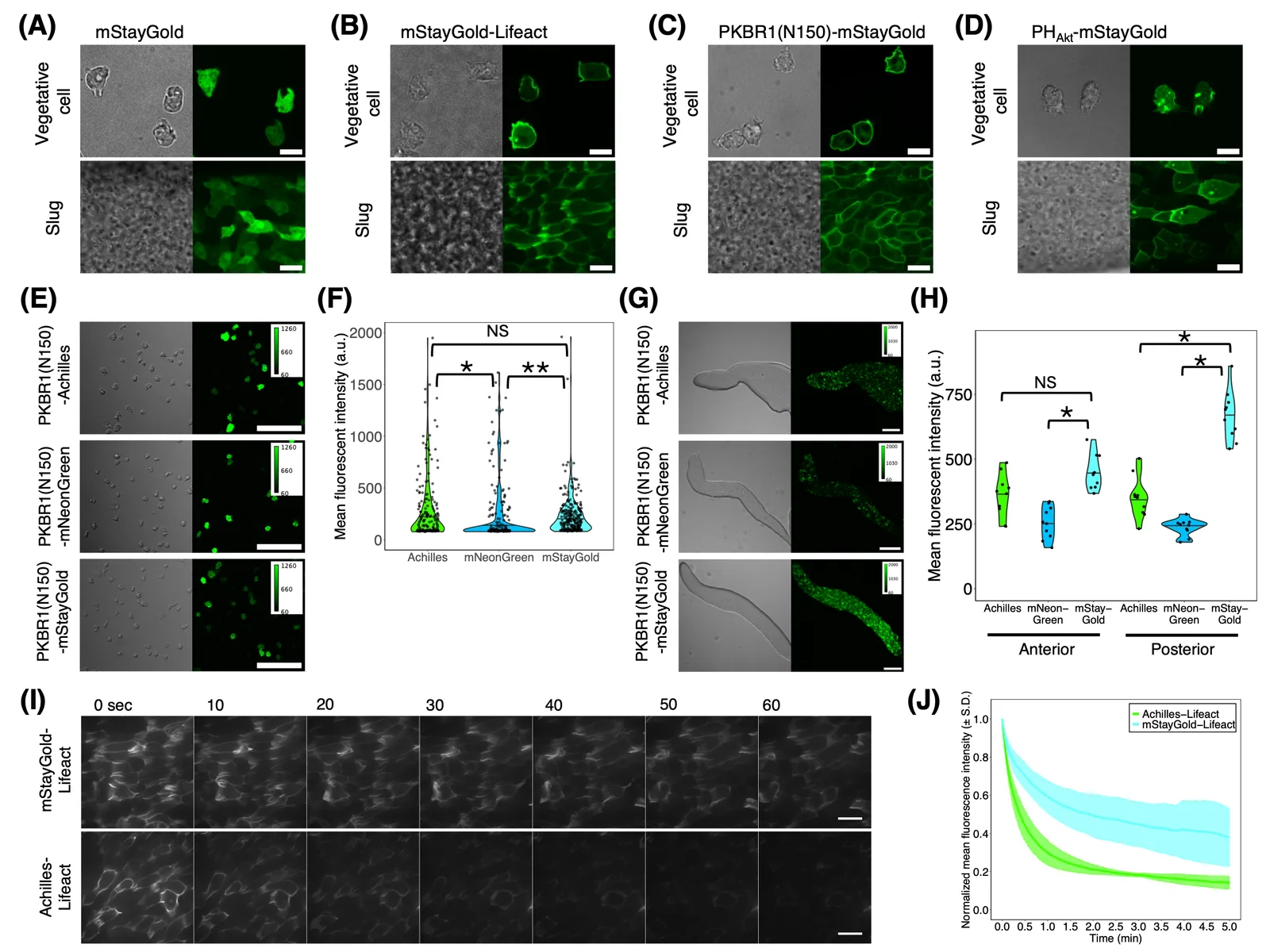



## Description

Fluorescent proteins are indispensable for microscopic imaging in cell and developmental biology, enabling one to capture dynamic processes at the molecular, cellular and tissue levels. AvGFP derived from *Aequorea victoria* (Chalfie et al., 1994) and its derivatives such as mClover3 (Bajar et al., 2016), cyan fluorescent protein mTurquoise2 (Goedhart et al., 2012) and the yellow fluorescent protein Venus (Nagai et al., 2002) have been widely used for live-cell imaging. In the cellular slime mold *Dictyostelium discoideum* , green fluorescent proteins, including AvGFP-derived GFP(S65T) (Heim et al., 1995) and Achilles (Yoshioka-Kobayashi et al., 2020), as well as mNeonGreen engineered from the *Branchiostoma lanceolatum* -derived LanYFP (Shaner et al., 2013), have been utilized for live-cell imaging of the cytoskeleton, intracellular signaling and cell differentiation (Aizawa et al., 2006; Hashimura et al., 2024; Nichols et al., 2020). However, the limited photostability of conventional fluorescent proteins can hinder applications requiring intense or prolonged excitation, such as super-resolution, high-speed, and long-term live-cell imaging.

The green fluorescent protein StayGold, derived from the *Cytaeis uchidae* CU17S protein (Hirano et al., 2022), offers a promising solution to these limitations. Compared with conventional green fluorescent proteins such as mNeonGreen and mClover3, StayGold exhibits exceptional resistance to photobleaching (Hirano et al., 2022). Although its native dimerization limits its use as a fluorescent tag for membrane-associated or oligomeric proteins, recent monomeric variants (Ando et al., 2024; Ivorra-Molla et al., 2024; Zhang et al., 2024) have addressed this issue. Among these, mStayGold (Ando et al., 2024) showed high brightness in living HeLa cells in a comparison with other monomeric StayGold variants (Shimozono et al., 2024) and maintained high brightness and photostability in zebrafish *Danio rerio* and nematode *Caenorhabditis elegans* (Ko & Mizumoto, 2025; Tschanz et al., 2025).

Here, to evaluate the applicability of mStayGold in *D. discoideum* , we expressed it as a stand-alone protein or as a fusion tag and examined its fluorescence using spinning-disc confocal microscopy. For comparison, each fluorescent protein was expressed using the same extrachromosomal vector backbone. During the vegetative and slug stages, mStayGold fluorescence was bright and uniformly distributed in the cytosol and the nucleus ( Fig. 1A ). mStayGold fused to Lifeact (Riedl et al., 2008) showed the expected localization pattern, exhibiting strong fluorescence at the cell cortex, pinocytic cups and the leading edges in vegetative cells ( Fig. 1B, upper panel). Similarly, in slug-stage cells, mStayGold-Lifeact localized to the cell cortex and the leading edges ( Fig. 1B, lower panel). mStayGold fused to the N-terminal sequence of PKBR1 containing a myristoylation signal (Meili et al., 2000) localized to the plasma membrane of both vegetative and slug-stage cells ( Fig. 1C ). mStayGold fused to the PH domain of Akt/PKB (Meili et al., 1999) localized to the pinocytic cups in vegetative cells and the front cell-cell contact sites in slug-stage cells ( Fig. 1D ). These localization patterns were comparable to those observed for mNeonGreen-Lifeact, PKBR1(N150)-Achilles and PH _Akt_ -Achilles (Hashimura et al., 2024).

These results indicate that mStayGold is a suitable alternative to other green and yellow fluorescent proteins such as mNeonGreen and Achilles. We next compared mStayGold expression in *D. discoideum* with that of Achilles and mNeonGreen when used as fusion tags. In vegetative-stage cells, the fluorescence of both PKBR1(N150)-Achilles and PKBR1(N150)-mNeonGreen appeared heterogeneous, with a subset of cells showing markedly higher intensity ( Fig. 1E, F). In contrast, PKBR1(N150)-mStayGold exhibited lower cell-to-cell variability in brightness ( Fig. 1E, F). The coefficients of variation were 1.08, 1.27, and 0.83 for Achilles, mNeonGreen, and mStayGold, respectively. A Fligner–Killeen test indicated a significant difference in variance among the three groups ( *P* < 10 ^-5^ ). The median population-level brightness of PKBR1(N150)-mStayGold was comparable to that of PKBR1(N150)-Achilles and higher than that of PKBR1(N150)-mNeonGreen ( Fig. 1E, F). In slugs, mNeonGreen fluorescence is known to be marked reduced with Achilles performing somewhat better (Hashimura et al., 2024) ( Fig. 1G ). In contrast, mStayGold fluorescence appeared stronger and thus more broadly visible across the slugs ( Fig. 1G ). The region-averaged fluorescent intensities of PKBR1(N150)-mStayGold for the anterior region was 76% and 25% higher than that of mNeonGreen and Achilles, respectively. In the posterior region, the corresponding increases were 181% and 90%, respectively ( Fig. 1H ).

Next, we studied the photostability of mStayGold in *D. discoideum* using spinning disk super-resolution microscopy (Hayashi & Okada, 2015). Time-lapse imaging was performed during the slug stage using cells expressing either mStayGold-Lifeact or Achilles-Lifeact. With image acquisition at 0.5-second intervals, Achilles-Lifeact fluorescence decreased by 70 % and became nearly undetectable after 1 minute. In contrast, mStayGold-Lifeact fluorescence decreased by 41 % and remained visible ( Fig. 1I, J; Movie 1). These results demonstrate the greater resistance of mStayGold to photobleaching than Achilles under intense illumination in *D. discoideum.*

Our results demonstrate robust mStayGold expression in *D. discoideum.* mStayGold fusion proteins displayed localization patterns consistent with those of Achilles and mNeonGreen. When expressed under a ubiquitous promoter, mStayGold exhibited more uniform fluorescence intensity in vegetative cells, which may reflect greater protein stability or lower toxicity in *D. discoideum* . The high mStayGold fluorescence intensity at the slug stage may also be advantageous for developmental studies. Furthermore, mStayGold’s greater resistance to photobleaching compared with Achilles makes it well suited for spinning-disk super-resolution microscopy, while the rapid maturation of Achilles (Yoshioka-Kobayashi et al., 2020) may offer complementary advantages for applications such as monitoring gene expression. Other potential applications in *D. discoideum* include high-speed imaging, single-particle tracking and long-term developmental imaging. Together, these properties highlight the utility of mStayGold for imaging dynamic cellular and developmental processes in *D. discoideum.*

## Methods

**Plasmids and transformation**

Plasmids and primers used in this study are summarized in the reagents section. To obtain N- and C-terminal fluorescent protein tag vectors (pDM326_ *act15p* :MCS-(GGS) _2_ -mStayGold and pDM326_ *act15p* :mStayGold-(GGS) _2_ -MCS), Dictyostelium codon-optimized DNA for mStayGold (Ando et al., 2024) was synthesized and inserted into the SpeI or BglII sites of pDM326 (Veltman et al., 2009) by fusing PCR-generated inserts and linearized vectors using the In-Fusion enzyme (In-Fusion Snap Assembly cloning kit, TAKARA, Shiga, Japan). The inserts were amplified using Phusion polymerase (New England Biolabs, Massachusetts, USA). The DNA sequence of codon-optimized mStayGold was as follows: ATGGCTAGTACAGGTGAAGAATTATTTACAGGTGTTGTTCCTTTCAAATTCCAATTAAAAGGCACAATTAATGGTAAATCATTCACTGTAGAAGGAGAAGGTGAAGGTAATAGTCATGAAGGATCACATAAAGGTAAATATGTATGTACATCAGGAAAATTACCAATGAGTTGGGCAGCATTAGGTACTTCTTTTGGATATGGTATGAAATATTATACCAAATACCCTTCAGGTTTGAAGAATTGGTTTCATGAAGTTATGCCAGAAGGTTTTACATATGATAGACATATACAATATAAAGGTGATGGTAGTATTCATGCTAAACATCAACATTTTATGAAGAATGGTACATATCATAATATCGTCGAGTTTACTGGTCAAGATTTTAAAGAAAATTCCCCAGTTTTAACAGGTGATATGGATGTATCTTTACCAAATGAAGTACAACATATTCCAATTGATGATGGTGTTGAATGTACTGTTACATTACAATATCCATTACTTTCTGATGAGTCAAAATGTGTTGAAGCATATCAAAATACTATTATAAAACCACTTCACAATCAACCAGCTCCAGATGTACCATTTCATTGGATAAGAAAACAATATACTCAATCAAAAGATGATACCGAAGAAAGAGATCATATTATTCAATCAGAAACATTGGAGGCCCATTTACCATGGCATGAACCATCAGCATCAGCAGTT.

To obtain expression vectors for Achilles-Lifeact and mStayGold-Lifeact, annealed Lifeact DNA oligos (Riedl et al., 2008) were inserted into the BglII and SpeI sites of #HH162 pDM326_ *act15p* :Achilles-(GGS) _2_ -MCS (Hashimura et al., 2024; Yoshioka-Kobayashi et al., 2020) and pDM326_ *act15p* :mStayGold-(GGS) _2_ -MCS, respectively. To generate pDM304_ *coaAp* :PKBR1(N150)-Achilles and pDM304_ *coaAp* :PKBR1(N150)-mNeonGreen, a fragment containing the *coaA* promoter and the coding sequence of PKBR1 (amino acids 1–150, containing a myristoylation motif) (Hashimura et al., 2024; Meili et al., 2000) was inserted into the XhoI and SpeI sites of #HH146 pDM304_ *act15p* :MCS-(GGS) _2_ -Achilles (Hashimura et al., 2024; Yoshioka-Kobayashi et al., 2020) and pDM304_a *ct15p* :MCS-mNeonGreen (Honda et al., 2021), respectively. To generate pDM304_ *coaAp* :PKBR1(N150)-mStayGold, a fragment containing (GGS) _2_ -mStayGold and the *act8* terminator was obtained from pDM326_ *act15p* :MCS-(GGS) _2_ -mStayGold by SpeI and HindIII digestion and inserted into the SpeI and HindIII sites of pDM304_ *coaAp* :PKBR1(N150)-mNeonGreen. To generate an expression vector for PH _Akt_ -mStayGold, a fragment containing the *coaA* promoter (Hashimura et al., 2024; Paschke et al., 2018) and the coding sequence of the PH domain of Akt/PKB (amino acids 1–111) (Hashimura et al., 2024; Meili et al., 1999) was inserted into pDM304, and a fragment of (GGS) _2_ -mStayGold was inserted into the SpeI site. For transformation, *D. discoideum* Ax4 cells were electroporated with 1 µg of plasmid DNA following the standard protocol (Nellen et al., 1984). Transformants were selected and maintained in modified HL5 growth medium containing 10 µg/mL G418 or 10 µg/mL Blasticidin S.

**Cell culture, development and labeling**

Modified HL5 medium was used for cell culture at 22 °C in either Petri dishes or shake flasks. The growth medium contained 10 µg/mL G418 or 10 µg/mL Blasticidin S where appropriate. To image the vegetative stage, cells were harvested, washed twice and resuspended in phosphate buffer (PB: 12 mM KH _2_ PO _4_ , 8 mM Na _2_ HPO _4_ , pH 6.5). An aliquot was plated directly onto a 24 × 50 mm coverslip (Matsunami, Osaka, Japan) to allow attachment to the glass surface before observation. For slug-stage imaging, washed cells were suspended at a density of 2 × 10 ^7^ cells/mL in PB, and 5 µL of the cell suspension was deposited on an agar plate (2% Bacto agar in Milli-Q water). After 18 h, agar pieces containing the slugs were excised and placed upside down onto a 25-mm-diameter round coverslip (Matsunami) mounted in a metal chamber (Attofluor, Invitrogen, Waltham, Massachusetts, USA) with or without a 50-µm-high spacer ring made of polyethylene terephthalate (vinyl patch transparent Ta-3N, Kokuyo, Osaka, Japan). The inner space of the ring was filled with liquid paraffin (Nacalai Tesque, Light 26132, Kyoto, Japan) prior to observation (Hashimura et al., 2019).

**Image acquisition and analysis**

Fluorescence images in Fig. 1A –D were acquired using an inverted microscope (IX83, Olympus/Evident Scientific, Tokyo, Japan) equipped with a 60× oil-immersion objective lens (UPlanSApo 60×, NA 1.35), a multibeam confocal scanning unit (CSU-W1, Yokogawa Electric, Musashino, Japan) and an electron-multiplying CCD camera (iXon 888, Andor, Belfast, Northern Ireland, UK). A 488 nm laser was used for excitation, together with a Di01-T405/488/568/647 dichroic beam splitter (Semrock, Rochester, NY, USA) and a 520/35 bandpass filter. To observe cells expressing PKBR1(N150) fusion probes in Fig. 1E –H, an inverted microscope (Ti-E, Nikon, Tokyo, Japan) equipped with a 20× objective lens (Plan Apo VC 20× DIC N2, NA 0.75) and a laser confocal point-scanning unit (A1R, Nikon) was employed. A 488 nm laser was used for excitation, together with a multiband 405/488/561 nm dichroic mirror and a 525/50 bandpass filter for detection. For super-resolution confocal imaging in Fig. 1I and J, an inverted microscope (IX83, Olympus/Evident Scientific, Tokyo, Japan) equipped with a multibeam confocal scanning unit (CSU-W1, Yokogawa Electric, Musashino, Japan), CMOS cameras (ORCA-Fusion BT, Hamamatsu Photonics, Hamamatsu, Japan) and a 100× oil-immersion objective lens (UPlanApo HR TIRF 100×, NA 1.50) was used in conjunction with a dedicated Nipkow disk and an intermediate magnification unit (IXplore SpinSR, Olympus/Evident Scientific, Japan). A 488 nm laser was used for excitation, together with a multiband dichroic mirror (T405/488/561/640 nm) and a 525/50 bandpass filter. The exposure time was 0.1 seconds.

Images were acquired in a 16-bit format, stored as TIFF files and analyzed using ImageJ and R. For quantitative measurements of the mean fluorescence intensity in individual cells, cell masks were obtained from brightfield images using Trainable Weka Segmentation (Arganda-Carreras et al., 2017). The mean fluorescence intensity of the cell regions was quantified per cell mask and the background signals were subtracted. To quantify the fluorescence signal in slugs, the mean fluorescence intensity of a 30 µm ^2^ rectangular region in the anterior or posterior region of each slug was measured. To quantify the fluorescence intensity profile from time-lapse images taken with the spinning-disc super-resolution microscope, the mean fluorescence intensity of the whole image area (1152 × 1152 pixels) was measured and normalized to the value at the first time frame. In statistical analyses, *P* values indicated in the figures were determined using the Wilcoxon rank-sum test with Bonferroni adjustment. In addition, for Fig. 1F, the Fligner–Killeen test was used to assess differences in variance among the groups, as described in the main text. The coefficient of variation was calculated as the standard deviation divided by the mean.

## Reagents

**Plasmids**

**Table:** 

Plasmid ID	Plasmid name	Description	Origin	Available from
–	pDM326	Empty vector	(Veltman et al., 2009)	NBRP-nenkin
#HH787	pDM326_ *act15p* :MCS-(GGS) _2_ -mStayGold-	extrachromosomal vector, C-terminal mStayGold tagging	This work	NBRP-nenkin
#HH643	pDM326_ *act15p* :mStayGold-(GGS) _2_ -MCS	extrachromosomal vector, N-terminal mStayGold tagging	This work	NBRP-nenkin
#HH748	pDM326_ *act15p* :mStayGold-Lifeact	extrachromosomal vector for expression of mStayGold-Lifeact	This work	NBRP-nenkin
#HH162	pDM326_ *act15p* :Achilles-(GGS) _2_ -MCS	extrachromosomal vector, N-terminal Achilles tagging	(Hashimura et al., 2024)	NBRP-nenkin
#HH586	pDM304_c *oaAp* :PKBR1(N150)-mStayGold	extrachromosomal vector for expression of PKBR1(N150)-mStayGold	This work	NBRP-nenkin
#HH584	pDM304_c *oaAp* :PH _Akt_ -mStayGold	extrachromosomal vector for expression of PH _Akt_ -mStayGold	This work	NBRP-nenkin
#HH146	pDM304_ *act15p* :MCS-(GGS) _2_ -Achilles		(Hashimura et al., 2024)	NBRP-nenkin
–	pDM304_ *act15p* :MCS-(GGS) _2_ -mNeonGreen		(Honda et al., 2021)	Sawai laboratory
#HH197	pDM304_c *oaAp* :PKBR1(N150)-Achilles	extrachromosomal vector for expression of PKBR1(N150)-Achilles	(Hashimura et al., 2024)	NBRP-nenkin
#HH122	pDM304_c *oaAp* :PKBR1(N150)-mNeonGreen	extrachromosomal vector for expression of PKBR1(N150)-mNeonGreen	(Honda et al., 2026)	NBRP-nenkin
#HH652	pDM326_ *act15p* :Achilles-Lifeact	extrachromosomal vector for expression of Achilles-Lifeact	This work	NBRP-nenkin

**Primers**

**Table:** 

Primer	Sequence (5′->3′)	Used for
mStayGold_fwd	AATAAAAATCAGATCCAAAAAATGGCTAGTACAGGTGAAG	#HH643
mStayGold-(GGS) _2_ _rev	AACTAGTACTAGATCTACTACCTCCTGAACCACCAACTGCTGATGCTGATGG	#HH643
(GGS) _2_ -mStayGold_fwd	CAGATCTAGTACTAGTGGTGGTTCAGGAGGTAGTGCTAGTACAGGTGAAG	#HH787
mStayGold _rev	TATTTATTTAACTAGATAAATGGGCCTCCAATG	#HH787
Lifeact_fwd	GATCTATGGGTGTCGCTGACCTGATAAAGAAGTTTGAAAGCATCTCCAAGGAAGAGA	#HH652, #HH 748
Lifeact_rev	CTAGTCTCTTCCTTGGAGATGCTTTCAAACTTCTTTATCAGGTCAGCGACACCCATA	#HH652, #HH 748

## Data Availability

Description: Time-lapse images of Achilles-Lifeact (left) and mStayGold-Lifeact (right) in slugs. Images were acquired at 0.5-second intervals for 5 minutes. Scale bar, 10 µm.. Resource Type: Audiovisual. DOI: https://doi.org/10.22002/kzm2f-2t774
